# Nordic biogas model in international contexts: Early-stage decision support for adaptation

**DOI:** 10.1177/0734242X241261998

**Published:** 2024-07-23

**Authors:** Roozbeh Feiz, Wisdom Kanda

**Affiliations:** Environmental Technology and Management, Department of Management and Engineering, Linköping University, Linköping, Sweden

**Keywords:** anaerobic digestion, technology adaptation, early-stage decision-making, sustainability benefits, decision-making guide, waste management

## Abstract

Global waste management challenges demand innovative and multi-functional solutions. The Nordic Biogas Model (NBM) based on anaerobic digestion of organic waste and valorization of its outputs provides several benefits beyond waste treatment such as energy recovery, nutrient recycling and climate impact mitigation. Despite these benefits, its international adoption remains limited, revealing an implementation gap. One way to address this gap is to adapt technology and knowledge from the provider to each specific context. This involves the embedding of the technology into the local context and the development of conditions such as formal and informal institutions over time. Based on decade-long interactions with Nordic companies and municipal decision-makers, we highlight the importance of communication between the technology provider and potential adopter, to ensure that the diverse sustainability benefits of NBM are acknowledged. Furthermore, most provider companies can benefit from a systematic guideline that supports early-stage decision-making as an essential component of the adaptation and implementation of the NBM in diverse international contexts. In this article, we offer suggestions for both: (1) how to better communicate the sustainability benefits of the NBM, and (2) how to assess the risk and opportunities of entering new markets at the early stages of decision-making.

## Introduction

More than 2 billion tonnes of waste are produced globally each year. This is projected to exceed 3 billion tonnes by 2050 (Kaza et al., 2018). Most of this waste, including the organic fractions of municipal solid wastes, is still landfilled or dumped. Worldwide, approximately 70% of waste is landfilled or is discarded in open dumps (Kaza et al., 2018). The large volume of waste and poor waste management in many parts of the world does not only put pressure on societies and the environment but also constitutes a significant loss of resources that could, in principle, be used more effectively based on, for example, circular economy principles.

Waste management is just one of many challenges facing societies, as highlighted by the UN’s Sustainable Development Goals, which span from regional issues like access to clean water and sanitation and affordable clean energy, to global concerns like climate change (United Nations, 2015). With around 600 cities globally having populations over one million (World Population Review, 2024), adopting a smart, multi-functional approach to waste management could simultaneously address various challenges. These solutions should avoid transferring problems or committing to unsustainable paths and could simultaneously tackle issues such as waste management, energy security, climate change, green public transport, urban pollution and foster a sustainable economy (Lindfors et al., 2019).

Biogas production through anaerobic digestion, representing a range of proven technologies can treat and valorise organic waste. This process facilitates organic waste management and generates biomethane, a renewable energy carrier with applications like natural gas but with a significantly lower climate impact (Liebetrau et al., 2022; Rama et al., 2023). Additionally, the residual digestate from this process serves as a nutrient-rich biofertilizer, closing the loop in nutrient recycling.

In the global context, biogas solutions have seen varying degrees of success, with the Nordic countries, especially Sweden and Denmark, exemplifying their effective implementation. Biogas production in these nations integrates into a broader ecosystem, encompassing waste management, energy production and agricultural practices. Sweden’s biogas production, closely linked with municipal waste management systems, systematically converts organic waste from numerous industries into biogas and biofertilizer – a model sometimes referred to as Nordic Biogas Model (NBM) (Lindfors et al., 2022). Meanwhile, Denmark has established numerous large-scale biogas plants as a core part of its strategy to reduce greenhouse gas emissions. These plants, often connected to the national gas grid, exemplify high-level system integration and resource efficiency. Such developments offer multifaceted benefits, such as reducing landfilling of organic waste, aiding climate change mitigation, boosting local energy security and supporting sustainable agriculture.

Despite numerous successful initiatives, such as the Nordic countries’ experiences with biogas solutions, and keen international interest in such multi-functional solutions, the international adoption of the NBM has been limited. Essentially, there are limited cases of the successful implementation of the NBM in international contexts which is also reflected by the very limited number of scientific articles. Analysing this topic has been an important motivation for the research reported in this article. This shortfall in widespread adoption foregoes dual benefits: enhancing domestic business capabilities and international standing for countries with successful experiences and offering significant waste management improvements for the receiving societies. This also underscores a notable ‘implementation gap’ in global waste management efforts in which socio-technical systems successfully implemented in one context face challenges in widespread adoption in other contexts. Overcoming the barriers to widespread adoption of multi-beneficial waste management solutions, such as NBM, could pave the way to bridge this implementation gap. It highlights the imperative for more unified efforts to close this gap and push forward global sustainability goals. In addition to the need to effectively communicate the multiple benefits of biogas solutions following NBM, a key barrier is the lack of systematic early-stage decision-making for the companies who provide biogas technology and knowledge. By early-stage we refer to a ‘pre-feasibility study’ phase in which some initial project ideas are formed, but resources are not yet committed to in-depth feasibility studies. Thus, in this article, we provide insights into addressing this implementation gap, especially at the early stages of communication and decision-making from the perspective of the biogas technology and knowledge providers.

## Key characteristics of the NBM

**Figure 1.**
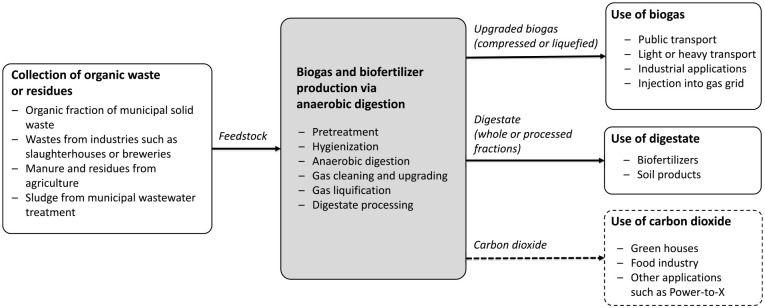
A simplified overview of the key characteristics of the Nordic Biogas Model.

Figure 1 depicts the NBM in a simplified overview as practised in the Nordic countries (e.g. see Feiz et al., 2022). The NBM is based on anaerobic digestion of organic waste, wastewater or low-grade biomass typically involving co-digestion of different types of feedstock. A common example is to use organic fraction of municipal solid waste (e.g. food waste) that depending on the local waste management system can be source-separated, or alternatively be separated at centralized facilities. The raw biogas produced is upgraded to biomethane for transport and other industrial applications. The residual digestate is used as biofertilizers or soil products while recent developments seek to capture and utilize carbon dioxide in, for example, green houses and food industry. Irrespective of the biogas system configuration, the defining characteristics of the NBM are built on the notion of multi-valorization through: (1) anaerobic digestion of organic waste, (2) upgrading of biogas into biomethane and (3) use of digestate as biofertilizers.

## Materials and methods

We used a combination of different research methods to collect and analyse empirical data as a basis for the insights presented in this article. The variety of actors and activities engaged in the Biogas Solutions Research Center (BSRC) made it an important platform for data collection and analysis. The BSRC is a Swedish national competence centre focusing on the development of resource efficient and effective biogas solutions. The centre adopts a transdisciplinary approach to create new knowledge through the interaction between several disciplines, and between researchers and practitioners. We collected empirical data and insights through more than a decade-long interactions with companies and municipal decision-makers. These interactions occurred through different activities and meeting occasions including annual conferences – something Van de Ven and Johson (2006) refer to as ‘engaged scholarship’.

Specifically, we arranged several workshops focused on the adaptation and dissemination of the NBM into international contexts. These workshops included the presentation of local conditions for the development of biogas systems such as the socio-economic (e.g. existing biogas related policies, waste sorting practices) and technological (e.g. substrate availability and distribution) situations in specific emerging economies such as Argentina, Brazil and South Africa. Another important aspect of our research method has been international field trips. These field trips offered the opportunity to meet and interact with the real problem owners (e.g. waste generators, biogas producers, biogas users and policymakers). Field trips have been essential to get an accurate and deeper understanding of the importance of the local pre-conditions and how that shapes the development of biogas systems. Finally, the BSRC has served as a platform for visiting international researchers and practitioners (e.g. companies, policymakers) from several countries. These exchanges have been important to cross fertilize insights from the local context with how the NBM has developed over time in Sweden. Altogether, these different approaches provide a rich empirical basis to synthesize insights on how to adapt and implement the NBM in an international context.

## Early-stage decision support for implementation of the NBM

**Figure 2.**
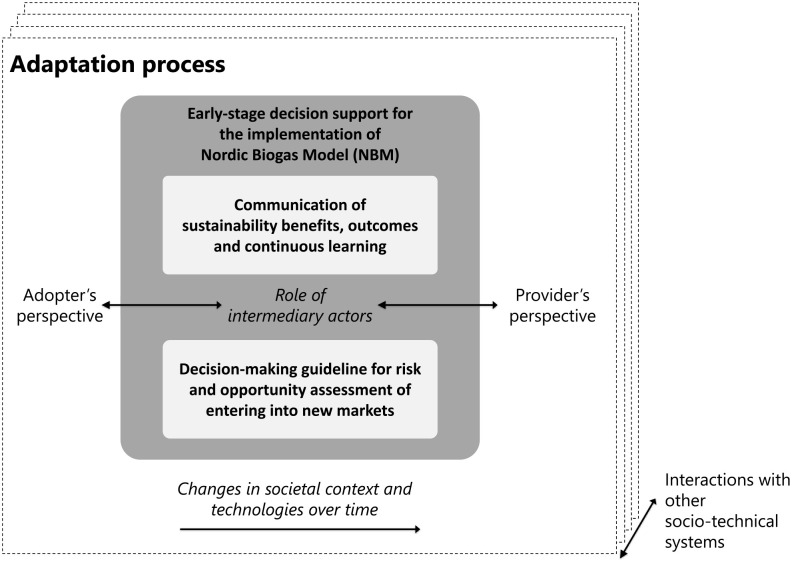
Overview of the adaptation process focusing on early-stage decision support including communication of sustainability benefits of NBM and decision-making guideline. NBM, Nordic Biogas Model.

The implementation of NBM in various international contexts extends beyond the basic ‘import/export model’ and is more accurately described as a process of adaptation. This adaptation process requires active participation from both adopters (e.g. a city council responsible for municipal waste management or public transport) and providers (e.g. a Nordic company active in the design, development and operation of biogas plants), often with the assistance of intermediary actors (i.e. organizations that connect adopters and providers with each other to realize biogas solutions, e.g. Swedfund International AB). Since biogas solutions based on the Nordic model are multi-sectoral, their implementation typically involves a diverse set of actors from agriculture, industry, civil society and also participation and support of citizens (e.g. in proper sorting of household waste) (Magnusson et al., 2022). It is a reflective process, mindful of the changing technological and societal conditions over time, and it interacts with other relevant socio-technical systems. Our emphasis here is on early-stages of decision making in which the role of provider, adopter and the intermediary as core actors is more pronounced. We provide insights to these actors for better communication of sustainability benefits of NBM as well as enhanced decision-making for entering a new market (Figure 2).

### Communication of sustainability benefits

Communicating the diverse effects of biogas solutions, especially those following the NBM, is crucial for enhancing the understanding and willingness among key actors in the receiving society (adopter) to actively pursue its implementation. The beneficial outcomes of these solutions are not limited to their primary functions of organic waste treatment, renewable energy generation and biofertilizer production. The advantages of biogas solutions extend beyond these core operations, encompassing a variety of direct and indirect effects (e.g. see Alberici et al., 2023). These multifaceted impacts necessitate a layered approach in communication strategies, which acknowledges the complexity and breadth of benefits associated with biogas systems (Table 1).

**Table 1.** Overview of our framework for communicating the potential sustainability benefits of successful implementation of the Nordic Biogas Model.

Impact area	Potential sustainability benefit
Energy recovery	Renewable energy recovery
	Local/regional energy security
	Local air quality improvement
	Less local noise
	Less acidification
Nutrient recovery	Renewable fertilizer production
	Nutrient and food security
	Agricultural soil fertility
	Sustainable farming practices
	Non-toxic agricultural soils and healthier food
	Reduced odour
Improved waste management	Cost-efficient waste and wastewater treatment
	Hygienization of biologically hazardous wastes
	Increased land availability
Climate impact mitigation	Reduction of greenhouse emissions
Broad sustainability implications	Improved regional economy and green jobs creation
	Improved public health
	Co-creation of knowledge

Source: Adapted from Feiz et al. (2024).

In comparison to organic waste management options such as incineration (energy recovery) or composting (nutrient recovery), biogas solutions offer the possibility of both energy and nutrient recovery and consequently many environmental, economic and social benefits (Feiz et al., 2024; Hagman and Eklund, 2016; Yamaji et al., 2024). This highlights the importance of moving beyond traditional cost–benefit analyses that focus solely on monetary or financial aspects. Instead, broader perspectives that include the environmental and social value of biogas solutions are essential (Yamaji et al., 2024). Furthermore, the success of these initiatives relies heavily on continuous communication, the dissemination of knowledge and learning processes. Such efforts are fundamental in cultivating a conducive environment for the adoption and expansion of NBM and other sustainable practices, underlining the necessity for ongoing engagement and education among stakeholders.

### A decision-making guideline

Many provider companies, such as Nordic firms with expertise in offering comprehensive system solutions, are often not large organizations with abundant resources. These companies may find it challenging to conduct detailed assessments of new opportunities for entering new markets, underscoring the importance of simple and effective methods for early-stage evaluation of the prerequisites for market expansion. Identifying key indicators and steps that can guide decision-making at this initial phase is crucial.

Our suggested guideline to early-stage decision-making (Table 2) focuses on identification of red flags or warning signs that might suggest potential challenges or risks in the new market. Many of such risks can be identified very early, even during the first contact (Step 1). The guideline also includes identifying opportunities that align with the company’s strengths and the unique needs of the target context. Only if the first contact assessment (Step 1) is positive, a preliminary assessment can be performed (Step 2) to establish a more complete view of the risks and opportunities of entering a new market.

**Table 2.** Overview of our early-stage decision-making guideline regarding entering a new market for the Nordic Biogas Model.

Step	Assessment area
Step 1: First contact assessment	
Case characterization and self-reflection	Adopter: potential client, country/region/city, type of problem that is expected to be addressed by the NBM, intended source of financing
	Raw materials: intended types and sources of feedstock, current treatment methods
	Use of products and by-products: intended application of produced biogas, digestate and carbon dioxide
	Expected offering (full, partial or customized NBM): source sorting, collection, anaerobic digestion, upgrading, liquification, digestate processing, etc. Relevance of offering to the existing system
	Self-reflection on the initial contacts: trustworthiness, competence, network association, entrepreneurship, cultural and personal aspects
Step 2: Preliminary assessment	
Preconditions for implementation	Feedstock potential: type, amount (available for NBM), quality
	Economy of the biogas system: revenues and share of different actors, costs and share of different actors
	Context and infrastructure: relevant infrastructure (road network, gas grid, . . .), source separation and collection, alternatives for waste management existing dependencies (e.g. is landfilling allowed and a cheap option?), major customers for gas and distances, major farming areas and distances, other required utilities (e.g. water and electricity supply)
	Financing of the project (provider’s perspective): revenues for the provider (from technology sale or offering), costs for the provider
	Provider’s risks, capabilities and strategies
	Partners and supporters: need for complementary capabilities, potential partner (both home and abroad), roles that partners should take
	Rules, regulations and standards: related to handling and treatment of feedstock, anaerobic digestion, use of products and by-products
	Knowledge, experience and attitude: adaptor’s familiarity with NBM, public perception, policy position on biogas

Source: Adapted from Kanda et al. (2021).

NBM, Nordic Biogas Model.

This guideline helps to (1) avoid investing significant resources in exploring a new market only to find out it was not a suitable option, and simultaneously (2) avoid missing out potentially good opportunities due to early-stage overcaution.

### Adaptation process

The adaptation of NBM to international markets requires a process tailored to the specific local context. The need for adaptation of biogas systems during their implementation has been called for in several previous studies (e.g. Zanatta, 2024). Adaptation processes are multifaceted and complex due to the multi-functional and cross-sectoral nature of biogas systems and the co-evolution between the technology and its context. Thus, the process of adaptation involves several actors and their processes which need to be coordinated by communication and collaboration across silos as Figure 2 suggests.

Since adaptation involves multiple local and international actors and their processes, there are often risks for gaps in communication and collaboration which can hinder the process. Intermediaries are found to bridge actors involved in situations where direct interaction is difficult due to several reasons including information asymmetry, power imbalances or cultural differences. However, the action of intermediaries goes beyond bridging in-between suppliers and adopters but also adapting the technology to the local context and standards. Thus, adaptation of NBM goes beyond just importation of biogas technologies: it requires local embedding through various levels of industrial and urban symbiosis (Chertow, 2000). Essentially, biogas solutions must be tailored to the local needs and integrated into the socio-economic conditions of the target country.

Furthermore, local and international actors need to be aware that adaptation of both the technology and the socio-economic context is a gradual process which takes time. It involves learnings, capacity building on issues such as policy, regulatory frameworks, standardizations efforts but also technological and infrastructure advancement. Essentially, adaptation is a reciprocal process between the system provider and potential adopters which fosters mutual learning and benefits (Figure 2). An essential part of the adaptation process is the need for early-stage assessment which provides insights regarding crucial adopter pre-conditions an essential input for decision making regarding resource commitments and system adjustments for international business development.

**Figure 3.**
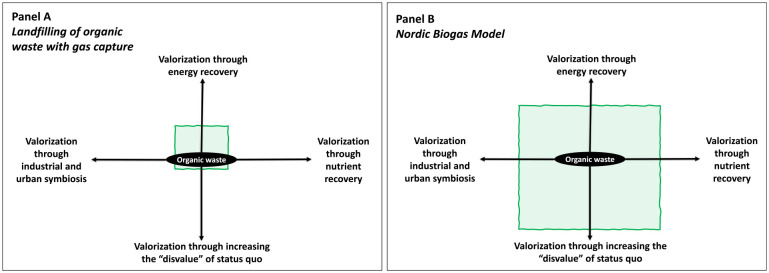
Multi-valorization possibilities of the NBM as a model for organic waste management. Panel A illustrates a limited and single-dimensional valorization of organic waste through landfill gas capturing. Panel B illustrates a multi-dimensional and broad valorization envelope through implementation of NBM. NBM, Nordic Biogas Model.

In summary, while technology providers bring valuable expertise and systems, they must be willing to adapt and evolve their expertise and technological systems based on local conditions, for example, feedback availability and quality, likewise the demands on local entities to adapt their practices and infrastructure. This holistic and reciprocal approach is essential for the adaptation of biogas systems. As shown in Figure 3, the valorization of organic waste and its outputs based on NBM does not only demand an adaptation of the technology for energy recovery and nutrient recirculation but also the development of framework conditions to provide incentives for valorization through increasing the ‘disvalue’ of maintaining the status quo. These framework conditions can exist in formal and informal institutions (e.g. waste sorting, biomethane quality standards, carbon tax, landfill taxes or bans, etc.) and facilitate experimentation, learning and collaboration through, for example, industrial symbiosis (Martin and Eklund, 2011). The framework conditions can either shrink or expand the valorization envelope depicted in Figure 3 and therefore have direct implications on how actors perceive risks and opportunities regarding the implementation of NBM.

Biogas system configurations based on maintaining the status quo such as landfilling of organic waste with gas capture can be considered as low hanging fruits and may not require systemic adaptations of the technology and its social dimensions. Such system configurations are often easier to implement but generate limited system value (Figure 3, panel A). More systemic adaptations of both the technology and its social dimensions are associated with biogas configurations that transform the wider adaptation context (Figure 3, panel B). These configurations including the NBM generate wider system benefits but can be slower and take time to develop since they require several deep structured changes.

Altogether, this article seeks to make three contributions. Firstly, we highlight that communicating the multiple sustainability benefits of NBM in a clear and compelling way is particularly important at an early stage to facilitate adoption process and to dissuade adopters to focus on narrow benefits and short-term returns. Secondly, due to context dependency and diversity of international markets it is important for technology providers to have a systematic approach for early-stage assessment of risks and opportunities. Thirdly, adopters and providers should be aware that adaptation of NBM involves navigating a complex landscape that requires coordination among stakeholders from various sectors – such as waste management, transport, agriculture and energy – which might have different logics, regulations and market conditions. The adaptation process often takes time and is gradual.

Overall, an integrative effort to communicate the multiple sustainability benefits of the NBM together with strategies to address its adaptation challenges is essential for enabling its sustainable implementation in international markets.

## Conclusion

To overcome the barriers in the international dissemination and implementation of the NBM, early-stage decision-making support which encompasses communication of the sustainability benefits and risk evaluations is crucial. Eliciting these aspects and addressing the relevant challenges would foster collaboration between technology providers and potential adopters and highlight the NBM as an important solution to global waste management challenges. Finally, our insights are not only relevant for biogas systems but also for the adaptation and dissemination of large technical systems in general in different contexts.
